# Upregulation of lncRNA BANCR associated with the lymph node metastasis and poor prognosis in colorectal cancer

**DOI:** 10.1186/s40659-017-0136-5

**Published:** 2017-10-02

**Authors:** Xiaogang Shen, Yifeng Bai, Bin Luo, Xiaogang Zhou

**Affiliations:** 1 Department of Gastrointestinal Surgery, Sichuan Academy of Medical Sciences and Sichuan Provincial People’s Hospital, 32 Second West Section of the First Ring Road, Chengdu, 610072 Sichuan China; 2 Department of Oncology, Sichuan Academy of Medical Sciences and Sichuan Provincial People’s Hospital, Chengdu, 610072 Sichuan China; 3 Department of Gastrointestinal Surgery, Sichuan Provincial People’s Hospital, 32 Second West Section of the First Ring Road, Chengdu, 610072 Sichuan China

**Keywords:** lncRNA, BANCR, Colorectal cancer, Lymph node metastasis, Prognosis

## Abstract

**Background:**

Growing evidence has supported that long non-coding RNAs (lncRNAs) could play vital roles in the development, progression, and prognosis of colorectal cancer (CRC). However, little is known about the clinical significance of BRAF-activated non-coding RNA (BANCR) in CRC. The aim of this study is to explore the clinical value of lncRNA BANCR in CRC patients.

**Methods:**

The expression of lncRNA BANCR was measured in 106 CRC tissues and 65 adjacent normal tissues using the quantitative real-time PCR.

**Results:**

The study showed that lncRNA BANCR was highly expressed in CRC tissues compared with adjacent normal tissues (*P* < 0.001). In addition, high expression of lncRNA BANCR was positively correlated with the lymph node metastasis (*P* < 0.001). Kaplan–Meier analysis showed that patients with high lncRNA BANCR expression had a shorter overall survival (OS) compared with the low lncRNA BANCR expression group (*P* = 0.001). Interestingly, for the group of patients with the lymph node metastasis, we found the similar result that high lncRNA BANCR expression was related to poor OS (*P* = 0.004). Furthermore, the multivariate Cox regression model analysis indicated that high expression of lncRNA BANCR was an independent poor prognostic factor in CRC patients (HR 2.24, 95% CI 1.22–4.16, *P* = 0.009).

**Conclusions:**

Upregulation of lncRNA BANCR may be associated with the lymph node metastasis and poor survival of CRC. LncRNA BANCR could be served as a novel and useful biomarker for CRC lymph node metastasis and prognosis.

## Background

Colorectal cancer (CRC) is the third most common malignant tumor and one of the leading causes of cancer-related deaths worldwide, making CRC a major public health problem all over the world [1]. According to 2015 Cancer Statistics, a total of estimated 3, 763,000 new CRC cases were diagnosed and 191,000 deaths resulted from the disease in China [2]. Although the mortality of CRC was dramatically decreased in recent decades as the effective therapeutic measures developed, such as surgical techniques, chemotherapy, and radiotherapy, the prognosis of CRC remains unsatisfactory because of tumor recurrence and metastasis [3, 4]. Thus, it is urgent to identify novel and useful biomarkers which can correlate to clinicopathological features, determine/evaluate prognosis, and provide new therapeutic strategies of CRC.

Long non-coding RNAs (lncRNAs), which are a class of non-coding RNAs with length more than 200 nucleotides, are being paid more and more attention due to its critical role in the transcriptional, epigenetic, and post-transcriptional regulation of gene expression [5, 6]. Recently, increasing evidence has suggested that lncRNAs could be involved in a variety of cellular processes, including cell cycle regulation, cell differentiation, proliferation, growth, and apoptosis [7–10]. In addition, lncRNAs have been demonstrated to be associated with various types of cancer, as tumor suppressors or oncogenes [11, 12]. Furthermore, a number of studies have investigated the aberrant expression of lncRNAs in CRC tissues and indicated that lncRNAs might serve as potential prognostic biomarkers and therapeutic targets [13–15].

The BRAF-activated non-coding RNA (BANCR), 693 bp in length and located on chromosome 9, is a newly functionally characterized lncRNA. Flockhart and his colleagues firstly observed that BANCR was overexpressed in melanoma cells and played a critical role in melanoma cell migration [16]. Subsequently, numerous studies have shown that BANCR could be involved in a plenty of biological processes, such as cell proliferation, migration, and invasion [17–19]. In addition, abnormal expression of BANCR has been investigated in various types of cancer such as gastric cancer, retinoblastoma, and non-small cell lung cancer [19–21]. However, the clinical value of BANCR in CRC is still unclear. In the present study, we investigated the expression of BANCR in CRC tissues and adjacent normal tissues. We then analyzed the relationship of BANCR expression with clinicopathological features, including the overall survival of CRC patients.

## Materials and methods

### Sample collection

The CRC patients hospitalized at the Sichuan Academy of Medical Sciences and Sichuan Provincial People’s Hospital in Chengdu (Chengdu, China) from June 2009 to May 2013 were consecutively recruited. The diagnosis of CRC was confirmed by a pathological examination combined with imaging examination (magnetic resonance imaging, MRI and/or computerized tomography, CT). Patients received treatments prior to radical surgical treatment were excluded. Finally, 106 CRC patients were enrolled. 106 freshly frozen colorectal cancer samples and 65 adjacent normal tissues were collected from the patients before any kind of therapeutic measures, and fresh samples were immediately preserved in liquid nitrogen. All patients were followed up by telephone calls every 3 months from the time of enrollment by personal or family contacts until death or the last time of follow-up. Finally, all the enrolled 106 CRC patients had complete follow-ups. The maximum follow-up time for the patients was 70.0 months (last follow-up in August 2015). During follow-up, 48 patients died from CRC, and the median survival time (MST) was 49.1 months. This study was approved by the ethics committee of Sichuan Academy of Medical Sciences and Sichuan Provincial People’s Hospital, and the written informed consent was also obtained from each participant after a clear explanation of study objective.

### Quantitative real-time transcription-PCR

Expression of lncRNA BANCR in CRC and adjacent normal tissues were measured. Total RNA was extracted from tissue samples using Trizol reagent (Invitrogen, San Diego, CA, USA) according to the manufacturer’s instructions. Total RNA from each sample was quantified and quality was verified using the NanoDrop ND-1000 spectrophotometer (Nanodrop, Wilmington, DE, USA). Total RNA (1 μg) was reverse-transcribed into complementary DNA (cDNA) with a sequence-specific primer and random hexamer primers using the Transcriptor First Strand cDNA Synthesis Kit (Roche, Penzberg, Germany). The sequence-specific primer for lncRNA BANCR was 5′-ACCATACCGAAACTTGAG-3′. The reverse transcription was performed at 37 °C for 15 min, then 96 °C for 5 s. The quantitative real-time PCR (qRT-PCR) was carried out using a Roche Light-Cycler (Roche, Basel, Switzerland) and SYBR Green reaction mix (Qiagen, Germany) to detect the level of lncRNA BANCR, with GAPDH mRNA as a normalizing control. The PCR primers for lncRNA BANCR or GAPDH mRNA were as follows: lncRNA BANCR forward: 5′-ACAGGACTCCATGGCAAACG-3′; lncRNA BANCR reverse: 5′-ATGAAGAAAGCCTGGTGCAGT-3′; GAPDH mRNA forward: 5′-ACCACAGTCCATGCCATCAC-3′; GAPDH mRNA reverse: 5′-TCCACCACCCTGTTGCTGTA-3′. The qRT-PCR amplification was performed in triplicate reactions under the following reaction conditions: (a) 96 °C for 10 min and (b) 40 cycles of 96 °C for 10 s, 67 °C for 15 s, and 85 °C for 20 s. The relative expression of lncRNA BANCR was calculated and normalized using the delta–delta CT (2^−ΔΔCt^) method relative to GAPDH mRNA. All qRT-PCR reactions were performed in triplicate. The median BANCR expression level was used as a cutoff value to define high or low BANCR expression.

### Statistical analysis

All statistical analyses were performed using SPSS 21.0 software (Statistical Package for the Social Sciences, Chicago, USA) and *P* < 0.05 was considered significant (two-tailed). The Paired-Samples *t* test was applied to test the differential expression of lncRNA BANCR in cancer tissues compared to adjacent normal tissues. The Chi square test was conducted for assessing differences in clinicopathological characteristics between two groups. The Kaplan–Meier method and log-rank test were used to plot the survival curves and compare the OS rates of patients with different lncRNA BANCR expression levels. The significance of survival variables was analyzed using the univariate and multivariate Cox regression model.

## Results

### LncRNA BANCR expression increased in CRC tissues

**Fig. 1:**
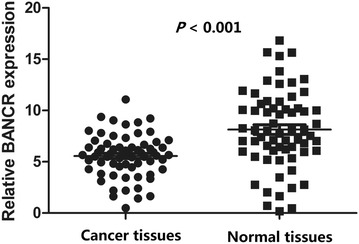
Expression level of lncRNA BANCR in CRC and normal tissues

In order to assess the effect of lncRNA BANCR in CRC, we firstly measured the expression levels of lncRNA BANCR in all 65 CRC tissues and 65 adjacent normal tissues. As shown in Fig. 1, lncRNA BANCR expression was significantly increased in cancer tissues compared with the paired adjacent normal tissues (*P* < 0.001).

### The relationship between lncRNA BANCR expression and clinicopathological features in CRC patients

To further investigate the relationship between lncRNA BANCR expression and CRC clinicopathological features, we then classified 106 CRC tissues into high and low lncRNA BANCR expression groups according to the median value of all samples (median ΔCT value = 7.89). As shown in Table 1, the results indicated that lncRNA BANCR expression levels in CRC significantly correlated with lymph node metastasis (*P* < 0.001). However, lncRNA BANCR expression did not correlate with other clinicopathological features, such as age (*P* = 0.430), gender (*P* = 0.558), tumor size (*P* = 0.556), differentiation (*P* = 0.672), venous invasion (*P* = 0.115), depth of invasion (*P* = 0.115), and location (*P* = 0.846).

**Table 1:** Correlation between lncRNA BANCR expression and clinicopathological factors of CRC

Variable	LncRNA BANCR expression (n, %)		χ^2^	*P* value
	Low (n = 53)	High (n = 53)		
Age (year)			0.622	0.430
≤60	29 (54.7)	33 (62.3)		
>60	24 (45.3)	20 (37.7)		
Gender			0.344	0.558
Male	28 (52.8)	31 (58.5)		
Female	25 (47.2)	22 (41.5)		
Tumor size (cm)			0.348	0.556
≤5	24 (45.3)	21 (39.6)		
>5	29 (54.7)	32 (60.4)		
Degree of differentiation			0.179	0.672
Well and moderate	36 (67.9)	38 (71.7)		
Poor	17 (32.1)	15 (28.3)		
Venous invasion			0.534	0.465
Negative	44 (83.0)	41 (77.4)		
Positive	9 (17.0)	12 (22.6)		
Depth of invasion			2.487	0.115
T1, T2	26 (49.1)	18 (34.0)		
T3, T4	27 (50.9)	35 (66.0)		
Lymph node metastasis			8.539	0.003
Absent	36 (67.9)	21 (39.6)		
Present	17 (32.1)	32 (60.4)		
Location			0.038	0.846
Colon	26 (49.1)	25 (47.2)		
Rectum	27 (50.9)	28 (52.8)		

### Upregulation of lncRNA BANCR associated with poor prognosis of CRC patients

**Fig. 2:**
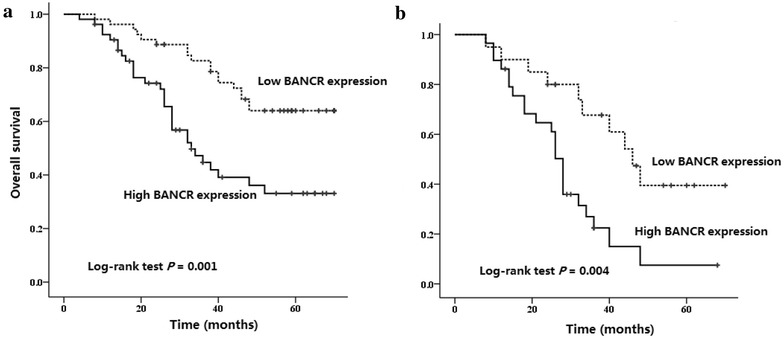
Kaplan–Meier survival curves of patients with CRC based on lncRNA BANCR expression. **a** Kaplan–Meier overall survival curves of patients with CRC based on BANCR expression. CRC patients in the high BANCR expression group had significantly lower overall survival rates than those in the low BANCR expression group (log-rank test *P* = 0.001). **b** Kaplan-Meier overall survival curves of CRC patients with the lymph node metastasis based on BANCR expression. CRC Patients with lymph node metastasis in the high BANCR expression group had significantly lower overall survival rates than those in the low BANCR expression group (log-rank test *P* = 0.004)

In order to evaluate the prognostic value of lncRNA BANCR expression in CRC patients, we finally conducted the Kaplan–Meier analysis and log-rank test to investigate the association between lncRNA BANCR expression levels and overall survival (OS) of CRC patients. As shown in Fig. 2, patients with high lncRNA BANCR expression levels displayed significantly lower OS rates than those with low lncRNA BANCR expression levels (*P* = 0.001). In the group of patients with lymph node metastasis, we found the similar result that the OS rates were significantly lower in patients with high lncRNA BANCR expression levels than those with low lncRNA BANCR expression levels (*P* = 0.004). However, this association was not found in the group of patients without lymph node metastasis (data not shown).

In addition, we used the univariate Cox regression model analysis and found that the tumor size (*P* = 0.012), depth of invasion (*P* = 0.025), lymph node metastasis (*P* < 0.001), and lncRNA BANCR expression (*P* = 0.001) were the prognostic factors of CRC patients (Table 2). Furthermore, the multivariate Cox regression model analysis indicated that high expression of lncRNA BANCR was an independent poor prognostic factor in CRC patients (HR 2.24, 95% CI 1.22–4.16, *P* = 0.009), regardless of tumor size, depth of invasion, and lymph node metastasis (Table 3).

**Table 2:** Univariate analyses of prognostic factors for overall survival of CRC patients

Variable	Patients, n (%)	Deaths, n (%)	HR (95% CI)	*P*
	N = 106	N = 48		
Age (year)				0.207
≤60	62 (58.5)	32 (66.7)	1	
>60	44 (41.5)	16 (33.3)	0.68 (0.37–1.24)	
Gender				0.888
Male	59 (55.7)	27 (56.3)	1	
Female	47 (44.3)	21 (43.8)	0.96 (0.54–1.70)	
Tumor size (cm)				0.012
≤5	45 (42.5)	18 (37.5)	1	
>5	61 (57.5)	30 (62.5)	2.19 (1.19–4.03)	
Degree of differentiation				0.372
Well and moderate	74 (69.8)	33 (68.8)	1	
Poor	32 (30.2)	15 (31.3)	1.32 (0.72–2.40)	
Venous invasion				0.542
Negative	85 (80.2)	37 (77.1)	1	
Positive	21 (19.8)	11 (22.9)	1.23 (0.63-2.42)	
Depth of invasion				0.025
T1, T2	44 (41.5)	17 (35.4)	1	
T3, T4	62 (58.5)	31 (64.6)	2.08 (1.10–3.94)	
Lymph node metastasis				<0.001
Absent	57 (53.8)	12 (25.0)	1	
Present	49 (46.2)	36 (75.0)	4.46 (2.39–8.33)	
Location				0.792
Colon	51 (48.1)	22 (45.8)	1	
Rectum	55 (51.9)	26 (54.2)	1.08 (0.61–1.91)	
LncRNA BANCR expression				0.001
Low	53 (50.0)	18 (37.5)	1	
High	53 (50.0)	30 (62.5)	2.74 (1.52–4.96)

**Table 3:** Multivariate analyses of prognostic factors for overall survival of CRC patients

Variable	Multivariate analysis		
	HR	95% CI	P
Tumor size (cm)			
>5 vs. ≤5	2.20	1.19–4.08	0.012
Depth of invasion			
T3-4 vs. T1-2	1.45	0.76–2.77	0.266
Lymph node metastasis			
Present vs. absent	4.26	2.24–8.10	<0.001
LncRNA BANCR expression			
High vs. low	2.24	1.22–4.16	0.009

## Discussion

To our best of knowledge, this is the first study to clarify the expression and clinical value of BANCR in CRC. In our findings, lncRNA BANCR was highly expressed in CRC tissues and positively correlated with the lymph node metastasis. In addition, high expression of lncRNA BANCR was an independent poor prognostic factor in CRC patients.

Up to now, increasing evidence has suggested that lncRNAs, the non-coding RNAs with the length more than 200 nucleotides, could be involved in CRC pathogenesis [22, 23]. For example, Niu and his colleagues investigated that 71 lncRNAs were up-regulated and 64 lncRNAs were down-regulated in CRC tissues compared with colorectal adenoma tissues, and lncRNA AK027294 closely correlated with colorectal cells proliferation, migration, and apoptosis [24]. Lian et al. found that overexpression of lncRNA HOTTIP was collated with an advanced pathological stage and a larger tumor size, and further functional analyses revealed that HOTTIP promoted CRC growth partially via silencing of p21 expression [25]. In addition, Zhao et al. demonstrated that plasma HOTAIR and CCAT1 could be used as a predictive biomarker for CRC screening [26]. Furthermore, another study reported that up-regulated lncRNA HOTAIR expression in primary tumors and in blood of CRC was associated with unfavorable prognosis of CRC, and HOTAIR blood levels could serve as surrogate prognostic biomarker in CRC [27]. However, not much was known about the clinicopathologic and prognostic significance of lncRNA BANCR expression in CRC.

Since Flockhart et al. firstly observed that BANCR was overexpressed in melanoma cells and knocking down BANCR expression could inhibit melanoma cell mobility [16], numerous studies had been conducted to explore the biological effects and clinical value in various types of cancer. For example, Zhang et al. reported that BANCR was highly expressed both in gastric tumor tissues and in cancer cells, and it could promote gastric cancer cells proliferation via regulation of NF-κB1 [17]. Meanwhile, Li et al. showed that BANCR was abnormally increased in both human malignant melanoma cell lines and tissues, and BANCR knockdown could significantly inhibit cancer cells proliferation by inactivating MAPK pathway [28]. In addition, Guo et al. similarly found that BANCR was frequently up-regulated in CRC tissues and cancer cell lines and significantly correlated with the lymph node metastasis and tumor stage of CRC [29]. Further study revealed that BANCR could induce the epithelial-mesenchymal transition through an MEK/extracellular signal-regulated kinase-dependent mechanism, and thus had the migratory effects [29]. These data suggested a tumor-promoting role of BANCR. However, other studies have shown the inconsistent results. One study found that down-regulation of BANCR could induce CRC cell growth in vitro and in vivo through the regulation of p21 protein [18]. Jiang et al. [30] and Sun et al. [21] both reported that knockdown of BANCR expression could promote lung cancer cells proliferation and migration in vitro. The differential expression and effects of BANCR in different types of cancer may be attributed to the variation of local tumor microenvironment and molecular pathways. In order to clarify the expression and clinical value of BANCR in CRC, we measured the expression levels of lncRNA BANCR in 65 pairs of CRC and adjacent normal tissues, and the results showed that lncRNA BANCR expression was significantly increased in cancer tissues compared with the paired adjacent normal tissues. Furthermore, we investigated the relationship between lncRNA BANCR expression and CRC clinicopathological features, and the results indicated that lncRNA BANCR expression levels in CRC significantly correlated with the lymph node metastasis.

So far, several studies also evaluated the prognostic value of lncRNA BANCR expression and suggested BANCR was an independent prognostic factor in several types of cancer. Li et al. detected the BANCR expression levels in 184 gastric cancer tissues and found that overexpressed BANCR was significantly associated with the clinical stage, tumor depth, lymph node metastasis and distant metastasis in gastric cancer patients, and further survival analysis indicated that up-regulated BANCR was an independent unfavorable prognostic factor of gastric cancer [20]. Su et al. also proved that high expression of BANCR was a poor independent prognostic factor for retinoblastoma patients by measuring 60 retinoblastoma samples [19]. However, Sun et al. [21] reported that down-regulated BANCR positively correlated with larger tumor size, advanced pathological stage, metastasis distance, and was an independent unfavorable prognostic factor of non-small cell lung cancer. In order to assess the prognostic value of BANCR expression in CRC patients, we further conducted the Kaplan–Meier analysis and multivariate Cox regression model analysis to find the correlation between BANCR expression levels and OS of CRC patients, and the results were consistent with Li et al. and Sun et al. CRC patients with high BANCR expression had a shorter OS, which meant high expression of BANCR was an independent poor prognostic factor in CRC patients.

In conclusion, our study provided the first evidence that upregulation of BANCR may be associated with the lymph node metastasis and poor survival of CRC. However, extensive functional researches and additional well-designed studies with different ethnic groups are warranted to confirm and extend our findings.

## Abbreviations

lncRNAs: long non-coding RNAs
CRC: colorectal cancer
BANCR: BRAF-activated non-coding RNA
OS: overall survival
MST: median survival time
qRT-PCR: quantitative real-time PCR

## References

[1] Siegel R, Naishadham D, Jemal A (2013). Cancer statistics, 2013. CA Cancer J Clin.

[2] Chen W, Zheng R, Baade PD (2016). Cancer statistics in China, 2015. CA Cancer J Clin.

[3] Baratti D, Kusamura S, Pietrantonio F, Guaglio M, Niger M, Deraco M (2016). Progress in treatments for colorectal cancer peritoneal metastases during the years 2010–2015. Syst Rev Crit Rev Oncol Hematol.

[4] Vatandoust S, Price TJ, Karapetis CS (2015). Colorectal cancer: metastases to a single organ. World J Gastroenterol.

[5] Ponting CP, Oliver PL, Reik W (2009). Evolution and functions of long noncoding RNAs. Cell.

[6] Tay Y, Rinn J, Pandolfi PP (2014). The multilayered complexity of ceRNA crosstalk and competition. Nature.

[7] Zhai W, Sun Y, Jiang M (2016). Differential regulation of LncRNA-SARCC suppresses VHL-mutant RCC cell proliferation yet promotes VHL-normal RCC cell proliferation via modulating androgen receptor/HIF-2alpha/C-MYC axis under hypoxia. Oncogene.

[8] Luo S, Lu JY, Liu L (2016). Divergent lncRNAs regulate gene expression and lineage differentiation in pluripotent cells. Cell Stem Cell.

[9] Wang X, Zhang W, Tang J (2016). LINC01225 promotes occurrence and metastasis of hepatocellular carcinoma in an epidermal growth factor receptor-dependent pathway. Cell Death Dis.

[10] Zhou J, Li M, Yu W (2016). AB209630, a long non-coding RNA decreased expression in hypopharyngeal squamous cell carcinoma, influences proliferation, invasion, metastasis, and survival. Oncotarget.

[11] Shao Y, Ye M, Jiang X (2014). Gastric juice long noncoding RNA used as a tumor marker for screening gastric cancer. Cancer.

[12] Yang Z, Guo X, Li G, Shi Y, Li L (2016). Long noncoding RNAs as potential biomarkers in gastric cancer: opportunities and challenges. Cancer Lett.

[13] Sun J, Ding C, Yang Z (2016). The long non-coding RNA TUG1 indicates a poor prognosis for colorectal cancer and promotes metastasis by affecting epithelial-mesenchymal transition. J Transl Med.

[14] Xie X, Tang B, Xiao YF (2016). Long non-coding RNAs in colorectal cancer. Oncotarget.

[15] Qiu JJ, Yan JB (2015). Long non-coding RNA LINC01296 is a potential prognostic biomarker in patients with colorectal cancer. Tumour Biol.

[16] Flockhart RJ, Webster DE, Qu K (2012). BRAFV600E remodels the melanocyte transcriptome and induces BANCR to regulate melanoma cell migration. Genome Res.

[17] Zhang ZX, Liu ZQ, Jiang B (2015). BRAF activated non-coding RNA (BANCR) promoting gastric cancer cells proliferation via regulation of NF-kappaB1. Biochem Biophys Res Commun.

[18] Shi Y, Liu Y, Wang J (2015). Downregulated long noncoding RNA BANCR promotes the proliferation of colorectal cancer cells via downregualtion of p21 expression. PLoS ONE.

[19] Su S, Gao J, Wang T, Wang J, Li H, Wang Z (2015). Long non-coding RNA BANCR regulates growth and metastasis and is associated with poor prognosis in retinoblastoma. Tumour Biol.

[20] Li L, Zhang L, Zhang Y, Zhou F (2015). Increased expression of LncRNA BANCR is associated with clinical progression and poor prognosis in gastric cancer. Biomed Pharmacother.

[21] Sun M, Liu XH, Wang KM (2014). Downregulation of BRAF activated non-coding RNA is associated with poor prognosis for non-small cell lung cancer and promotes metastasis by affecting epithelial-mesenchymal transition. Mol cancer.

[22] Xu MD, Qi P, Du X (2014). Long non-coding RNAs in colorectal cancer: implications for pathogenesis and clinical application. Modern Pathol Off J US Can Acad Pathol Inc.

[23] Han D, Wang M, Ma N, Xu Y, Jiang Y, Gao X (2015). Long noncoding RNAs: novel players in colorectal cancer. Cancer Lett.

[24] Niu H, Hu Z, Liu H (2016). Long non-coding RNA AK027294 involves in the process of proliferation, migration, and apoptosis of colorectal cancer cells. Tumour Biol.

[25] Lian Y, Ding J, Zhang Z (2016). The long noncoding RNA HOXA transcript at the distal tip promotes colorectal cancer growth partially via silencing of p21 expression. Tumour Biol.

[26] Zhao W, Song M, Zhang J, Kuerban M, Wang H (2015). Combined identification of long non-coding RNA CCAT1 and HOTAIR in serum as an effective screening for colorectal carcinoma. Int J Clin Exp Pathol.

[27] Svoboda M, Slyskova J, Schneiderova M (2014). HOTAIR long non-coding RNA is a negative prognostic factor not only in primary tumors, but also in the blood of colorectal cancer patients. Carcinogenesis.

[28] Li R, Zhang L, Jia L (2014). Long non-coding RNA BANCR promotes proliferation in malignant melanoma by regulating MAPK pathway activation. PLoS ONE.

[29] Guo Q, Zhao Y, Chen J (2014). BRAF-activated long non-coding RNA contributes to colorectal cancer migration by inducing epithelial-mesenchymal transition. Oncol Lett.

[30] Jiang W, Zhang D, Xu B (2015). Long non-coding RNA BANCR promotes proliferation and migration of lung carcinoma via MAPK pathways. Biomed Pharmacother.

